# Surgery for Gynecomastia in the Islamic Golden Age: *Al-Tasrif* of Al-Zahrawi (936–1013 AD)

**DOI:** 10.5402/2012/934965

**Published:** 2012-09-20

**Authors:** Seyed Hadi Chavoushi, Kamyar Ghabili, Abdolhassan Kazemi, Arash Aslanabadi, Sarah Babapour, Rafail Ahmedli, Samad E. J. Golzari

**Affiliations:** ^1^Hematology and Oncology Research Center, Tabriz University of Medical Sciences, Tabriz 51656-65811, Iran; ^2^Physical Medicine and Rehabilitation Research Center, Tabriz University of Medical Sciences, Tabriz 51656-65811, Iran; ^3^Medical Philosophy and History Research Center, Tabriz University of Medical Sciences, Tabriz 51656-65811, Iran; ^4^Students' Research Committee, Tabriz University of Medical Sciences, Tabriz 51656-65811, Iran; ^5^Institute of Philosophy, Sociology and Law, Azerbaijan National Academy of Sciences, 1141 Baku, Azerbaijan; ^6^Tuberculosis and Lung Disease Research Center, Tabriz University of Medical Sciences, Tabriz 51656-65811, Iran

## Abstract

The rise of European science during the Renaissance is greatly indebted to the flourishing of the sciences during the Islamic Golden Age. However, some believe that medieval Islamic physicians and in particular surgeons had been merely a medium for Greco-Roman ideas. Contrarily, in some medieval Islamic medical books, such as *Al-Tasrif* of Al-Zahrawi (936–1013), the surgical instructions represent a change in the usual techniques or are accompanied by a case history, implying that the procedure was actually undertaken. Along with the hundreds of chapters on different diseases and related medical and surgical treatments, *Al-Tasrif* includes a chapter on surgical techniques for gynecomastia. The present paper is a review of the description of the surgical management of gynecomastia by Al-Zahrawi as well as that of the ancient Greek, medieval, and modern medicine. Although Al-Zahrawi seemed to base his descriptions of surgery for gynecomastia upon those of Paulus of Aegina, his modification of the procedure and application of the medicinal substances might be indicative of Al-Zahrawi's own practice of the procedure. Al-Zahrawi's surgical procedures remained unchanged for many centuries thenceforward until the technological evolution in the recent centuries.

## 1. Introduction

 The rise of European science during the Renaissance is greatly indebted to the flourishing of the sciences during the Islamic Golden Age [1–3]. In the Eastern Caliphate of Baghdad, Muslim scholars translated and assimilated the Greek works, while adding their own commentaries [4]. Thereafter, thanks to their own perceptive observations, trials, and skills, renowned scholars such as Muhammad ibn Zakariya al-Razi or Rhazes (865–925), Ali ibn al-Abbas al-Majusi or Haly Abbas (930–994), and Abu-Ali al-Husain ibn Abdollah ibn Sina or Avicenna (981–1037) remarkably contributed to the scientific treasure of this era [5]. Meanwhile, in the Western Caliphate of Córdoba, Muslim physicians and philosophers almost as brilliant as those of the East strongly promoted this scientific movement [6]. Studied and practiced medicine at Seville and Córdoba, Al-Zahrawi or Albucasis (936–1013), Ibn Zuhr or Avenzoar (1092–1162), and Ibn Rushd or Averroës (1126–1198) were the most influential physicians of the western lands [7].

 Nonetheless, some believe that medieval Islamic physicians had been merely a medium for Greco-Roman ideas. On the other hand, Abbasids' attempts to resurrect the conviction that the Greek medicine is in essence derived from Persian have persuaded some medical historians to repudiate the former idea [8]. The idea of Muslim physicians being solely interpreters of the science rather than compilers is mostly attributed to the medieval Islamic surgeons [9, 10]. In particular, where the Islamic surgeon neglected to indicate an example of a procedure's usage, or to modify the procedure or instrumentation in any way from that handed down from his predecessors, such an interpretation of a literary tradition unrelated to the actual practice of surgery is encouraged. In contrast, in some medieval Islamic medical books, the surgical instructions represent a change in the usual techniques or are accompanied by a case history, implying that the procedure was actually undertaken [8]. A proof of the latter is the surgical instruments first depicted in details by Al-Zahrawi in his book of *Al-Tasrif* [11]. Herein, we review the description of the surgical management of gynecomastia by Al-Zahrawi and compare it with that of the ancient Greek, medieval, and modern medicine.

## 2. Outline Biography

**Figure 1:**
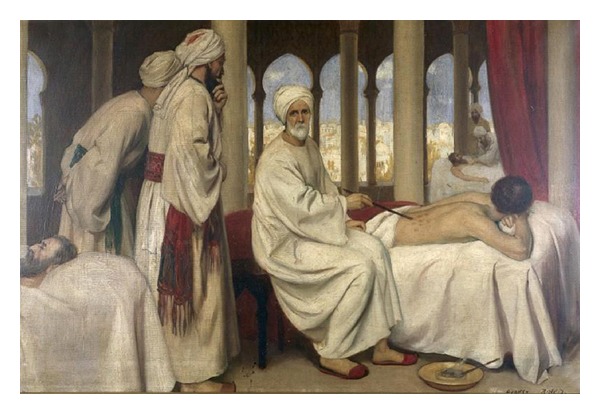
Al-Zahwari blistering a patient in the hospital at Córdoba whiles his students looking on. Reproduced with permission from Wellcome Library, London.

 Abul Qasim Khalaf ibn al-Abbas al-Zahrawi, known as Abulcasis or Albucasis in the West (Figure 1), was born in al-Zahra (near Córdoba, Spain) in 936 AD [12, 13]. He sprang from the Ansar tribe of Medina, Saudi Arabia, who had settled earlier in Spain [14]. He lived most of his life in Córdoba where he studied, taught, and practiced medicine and surgery [12, 15]. Al-Zahrawi became one of the most famous surgeons of the Muslim era and was physician to Abd al-Rahman III (912–961) and his son Al-Hakam II (915–976) of Spain, the Umayyad Caliphs of Córdoba [16]. Unlike his well-known published work, a few details remain about Al-Zahrawi's life. Al-Zahrawi was first mentioned by the Andalusian scholar, Abu Muhammad ibn Hazm (993–1064), as one of the great physician surgeons of the Moorish Spain [12, 13]. The first known biography of Al-Zahrawi was illustrated in Al-Humaydi's *Jadhwat al-Muqtabis fi Dhikri Volat al-Andalus (On Andalusian Savants) *compiled six decades after Al-Zahrawi's death [12, 13]. After nearly five decades of medical career full with great original contributions particularly in the court of Caliph, Al-Zahrawi died in 1013 AD [17, 18].

## 3. *Kitab Al-Tasrif*

**Figure 2:**
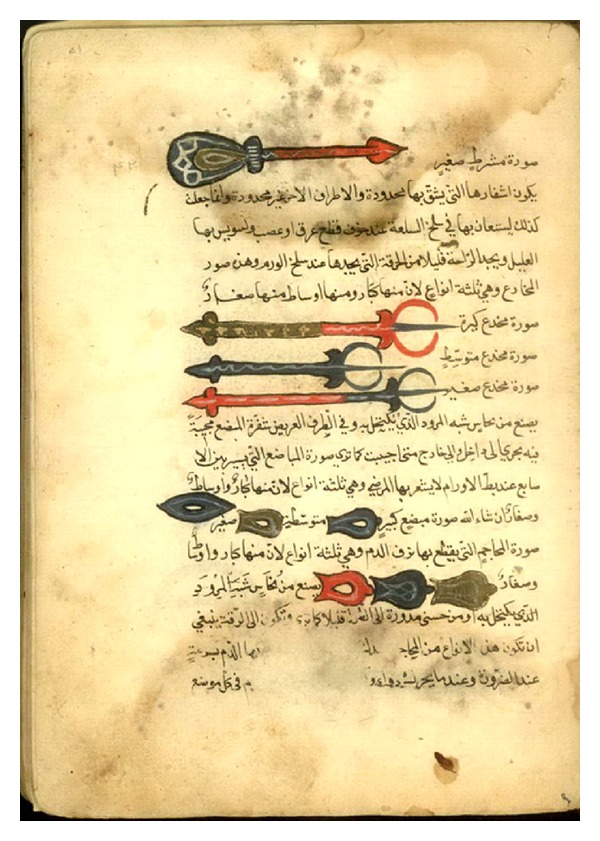
A page of *Al-Tasrif* depicting the surgical tools devised or utilized by Al-Zahrawi (Sana Library version). Reproduced with permission from Library, Museum and Document Center of Iran Parliament, Tehran.

 Al-Zahrawi's thirty-book medical treatise, *Kitab al-Tasrif Leman Ajiz an al-Taalif* (*The Arrangement of Medical Knowledge for One who is not Able to Compile a Book for Himself*), completed in 1000 AD, covers a broad range of medical topics [15, 19]. *Al-Tasrif*, an illustrated encyclopedia of medicine and surgery, greatly influenced the progress of medicine and surgery in Europe since it was translated into Latin by Gerard of Cremonia (1114–1187), an Italian translator of Arabic scientific works [14]. Thanks to translations into different European languages including French and English, *Al-Tasrif* displaced Avicenna's *The Canon of Medicine* as the textbook for medical education in many of the European universities between 12th–17th centuries AD [15, 19]. The most important part of *Al-Tasrif* comprises three books on surgery: on cauterization, on incision, perforation, venesection, and wounds, and on bone-setting [12]. These books contain various aspects of surgical treatment in details based on Al-Zahrawi's personal experiences of the surgical operations (Figure 2) [15]. Al-Zahrawi based his published work, in particular the surgical books, upon Greek authorities, of whom the most impressing was Paulus of Aegina (ca. 625–690). Nonetheless, Al-Zahrawi added his several personal experiences along with almost 200 illustrations of instruments' designs. The latter, many of which Al-Zahrawi devised himself, is deemed as an important innovation in the history of surgical literature [8]. In later centuries, almost all European surgeons referred to Al-Zahrawi's masterpiece of surgery [20]. Interestingly, Şerafeddin Sabuncuoğlu (1385–1468), a medieval Ottoman surgeon and physician, compiled a translation of Al-Zahrawi's *Al-Tasrif* in his book of *Cerrahiye-tu l-Hanniyye* (*Imperial Surgery*) accompanied with his own experiences and illustrations of surgical procedures [21]. The second surgical book of *Al-Tasrif* includes a chapter on surgical treatment of gynecomastia, “On the treatment of male breast when it resembles the female.” What follows is a translation of the chapter related to the gynecomastia and the related surgical treatment.

## 4. From *Kitab Al-Tasrif*

**Figure 3:**
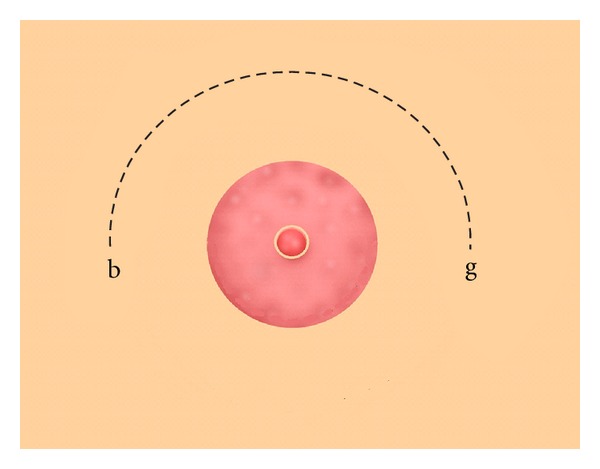
Surgical treatment of moderate gynaecomastia according to Al-Zahrawi. A lunate incision is made above the breast and the subcutaneous fat is removed (points b and g delimit the incision).

**Figure 4:**
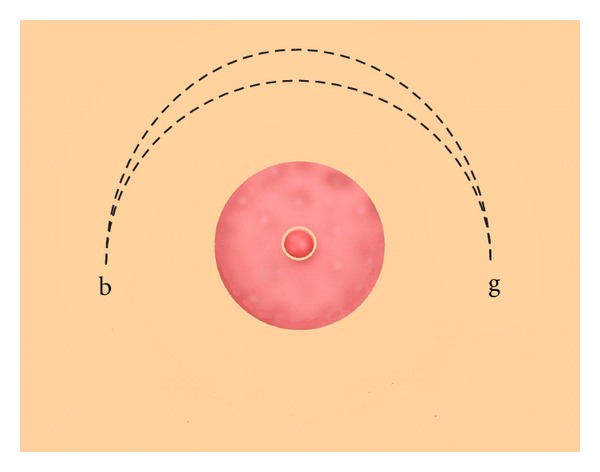
Surgical treatment of severe gynaecomastia according to Al-Zahrawi. Two lunate incisions are made along the upper part of the breast to allow the removal of subcutaneous fat along with the excess skin (points b and g delimit the incision).

 The breast of some males may swell on the attaining puberty so as to resemble the female breast, and they remain permanently swollen and ugly. If this is abhorrent, a semicircular incision should be made on the breast like the figure from b to g (Figure 3), then dissect away all the fatty tissue and pack the wound with a cicatrizing compound and sew the lips together and dress until healed. But if the breast is pendulous and flabby on account of its size, as happens in women, you should make two semicircular incisions on the upper side the ends joining one another, in such wise that the longer incision encircles the other like this (Figure 4), from b to g. Then dissect away the skin between the two incisions and remove the fat and sew up as we have described, and apply styptic powder and the necessary dressings until healed. But if you cannot make incision as full and perfect as it ought to have been, because the patient is restless, or because hemorrhage arises, you should pack the wound with cotton wool soaked in corrosive ointment and leave it till it eats away the reminder of the fat, then dress until healed [12]. 

## 5. Discussion

 Although the concept of breast enlargement in males was first implied by Aristotle (384–322 BC), the well-known Roman physician Claudius Galenus (129–200 AD) coined the term “gynecomastia” in the second century AD [30]. Thereafter, “gynecomastia” has been applied to an atypical enlargement of male's breast. There is no historical evidence of any surgical treatment for gynecomastia in pre-Galenic, Galenic, and post-Galenic era until Paulus of Aegina, a seventh century Byzantine Greek physician, first indicated the surgical treatment of gynecomastia in his *Epitome of Medicine* (*Seven Books*) [31]. Later in the Islamic Golden Age, Haly Abbas described surgical management of gynecomastia in his *Kitab al-Maliki* (*The Royal Book*) mostly based on that of Paulus of Aegina [32]. Another Muslim physician attempted to provide the physicians and surgeons with surgical treatment of gynecomastia was Al-Zahrawi or Albucasis, a well-known Andalusian surgeon. Although some believe that Al-Zahrawi merely replicated the writings on gynecomastia from Paulus of Aegina's Epitome [32], Al-Zahrawi's modification of the procedure and administered medications (see below) might be indicative of his own practice of the procedure. Almost four centuries later, Şerefeddin Sabuncuoğlu illustrated the surgical techniques for the management of gynecomastia quite based on the related descriptions by Paulus of Aegina and Al-Zahrawi [33].

 The surgical techniques used to treat the gynecomastia throughout the medieval era were roughly the same. Al-Zahrawi described two different surgical techniques for the treatment of gynecomastia. The first technique included making a lunate incision above the breast, removal of the subcutaneous fat, and application of a cicatrizing compound [12]. In contrast to the site of incision (below the breast) indicated by Paulus of Aegina [34], Al-Zahrawi recommended making the incision above the breast. Al-Zahrawi's different techniques might provide further breast uplift. On the other hand, unlike his Greco-Roman predecessors, Al-Zahrawi suggested usage of the flesh-growing substance at the end of the surgical procedure for the management of gynecomastia. As later stated by Ibn Sina or Avicenna (980–1037 AD) in the *Canon of Medicine*, dragon's blood (*Calamus draco*) is an example of the cicatrizing or flesh-growing substance [35]. Interestingly, wound healing and antimicrobial effects of this medicinal herb have been recently proved by the modern medicine [22]. This might imply that Al-Zahrawi was not only knowledgeable about the surgical techniques but also familiar with pharmacology [36].

 The second surgical technique described by Al-Zahrawi for the treatment of gynecomastia involved making two lunate incisions along the upper part of the breast to allow the removal of subcutaneous fat and redundant skin as well as application of styptic powder [12]. Although the former was a verbatim description of Paulus of Aegina's surgical technique for the management of severe gynecomastia [32], Al-Zahrawi first recommended application of the styptic powder following the severe gynecomastia surgical procedure. According to the traditional Islamic medicine, sprinkling powder over the surgical wound accelerates the wound healing and consists of aloe (*Aloe vera*), dragon's blood (*Calamus draco*), gum Arabic tree (*Acacia arabica*), sarcocolla (*Astrargalus fasciculifolius bioss*), and myrrh (*Commiphora molmol*) [37]. The efficacy of the natural substances prescribed by Al-Zahrawi for the gynecomastia surgical wound healing has been confirmed in the modern medicine (Table 1).

 It is now believed that hematoma is the most common complication of surgery for the relief of gynecomastia. Therefore, careful hemostasis and compression dressings are used to minimize this complication [38]. It is noteworthy that Al-Zahrawi lucidly indicated hemorrhage as the complication of the surgical management of gynecomastia and, therefore, he recommended compression cotton dressing [12]. This important detail of the surgery for the treatment of gynecomastia had not been previously reported by the ancient Greek, Roman, and even other medieval Islamic physicians and surgeons. In addition, Al-Zahrawi recommended application of corrosive ointment to erode the reminder of the fat. According to the traditional Islamic medicine resources, *zanjar* (verdigris or copper acetate) was a well-known corrosive agent [35, 39]. Verdigris and other copper-containing compounds were administered for the treatment of inflammatory diseases in the ancient Egyptian and Roman medicine [40]. In the history of gynecomastia, Al-Zahrawi was the first physician and surgeon who employed the verdigris in the treatment of some cases of gynecomastia. The efficacy of this substance might be attributed to its anti-inflammatory activity [41].

 In modern practice, surgical management of gynecomastia is recommended after any underlying causes have been treated or following the failure of pharmacologic treatment [38]. In contrast, the medieval clinicians including Paulus of Aegina and Al-Zahrawi neglected the probable etiologies of the condition in their writings, albeit they were pioneers in clinical endocrinology [42]. This might give rise to the most of the medieval Islamic physicians' failure to indicate the gynecomastia in their medical books. In modern medicine, all related surgical procedures are designed to completely remove the excess breast tissue while minimizing scarring on the chest. As a result, semicircular intraareolar incision together with liposuction constitutes the treatment of mild to moderate gynecomastia, while more severe cases require periareolar incision and subsequent skin resection and nipple transposition [38]. In comparison with the modern aesthetic criteria, the aesthetic outcome of Al-Zahrawi's surgical techniques for the treatment of gynecomastia, in particular with respect to the shape and site of the surgical incision and excess skin resection, might be considered as satisfactory but not excellent, at least for the time being.

 The breast reduction for men with gynecomastia was first introduced in Byzantium. Later, although Al-Zahrawi seemed to base his descriptions of surgery for gynecomastia upon those of Paulus of Aegina, his modification of the procedure and application of the medicinal substances might be indicative of Al-Zahrawi's own practice of the procedure. Al-Zahrawi's surgical procedures remained unchanged for many centuries thenceforward until the technological evolution in the recent centuries.

## Figures and Tables

**Table 1:** Natural substances and their confirmed effects in modern medicine described in *Al-Tasrif* for the surgical management of gynecomastia.

Common name	Scientific name	Effects
Dragon's blood	*Calamus draco*	Wound healing [22], antimicrobial [22]
Sarcocolla	*Astragalus fasciculifolius*	Wound healing [23, 24]
Myrrh	*Commiphora molmol*	Wound healing [25], antibacterial [26]
Aloe	*Aloe vera*	Antimicrobial [27], wound healing [28]
Gum Arabic tree	*Acacia arabica*	Antibacterial [29]

## References

[1] Elgood C (1951). *A Medical History of Persia and the Eastern Caliphate. From the Earliest Times Until the Year 1932*.

[2] Khodadoust K, Ardalan M, Ghabili K, Golzari SE, Eknoyan G Discourse on pulse in medieval Persia-the Hidayat of Al-Akhawayni (?-983AD).

[3] Hosseini SF, Alakbarli F, Ghabili K, Shoja MM (2011). Hakim Esmail Jorjani (1042-1137ad): persian physician and jurist. *Archives of Gynecology and Obstetrics*.

[4] Ardalan MR, Shoja MM, Tubbs RS, Eknoyan G (2007). Diseases of the kidney in medieval Persia—the Hidayat of Al-Akawayni. *Nephrology Dialysis Transplantation*.

[5] Golzari SE, Khodadoust K, Alakbarli F (2012). Sleep paralysis in medieval Persia—the Hidayat of Akhawayni (?-983 AD). *Journal of Neuropsychiatric Disease and Treatment*.

[6] Abdel-Halim RE (2005). Contributions of Ibn Zuhr (Avenzoar) to the progress of surgery. A study and translations from his book Al-Taisir. *Saudi Medical Journal*.

[7] Golzari SE, Mirinejad MM, Kazemi A, Khalili M, Ghabili Avenzoar K (1092-1162 AD) and Averroes (1126-1198 AD): andalusian Muslim physicians.

[8] Pormann PE, Savage-Smith E (2007). *Medieval Islamic Medicine*.

[9] Savage-smith E (1995). Attitudes toward dissection in medieval islam. *Journal of the History of Medicine and Allied Sciences*.

[10] Shoja MM, Tubbs RS (2007). The history of anatomy in Persia. *Journal of Anatomy*.

[11] Anis I, Khan AB (1984). Surgery in the medieval Muslim world. *Indian Journal of History of Science*.

[12] Spink MS, Lewis GL (1973). *Albucasis on Surgery and Instruments*.

[13] Al zahrawi HR (2006). Father of surgery. *Heart Views*.

[14] Shaikh I http://www.muslimheritage.com.

[15] Nabri IA (1983). El Zahrawi (936-1013 AD), the father of operative surgery. *Annals of the Royal College of Surgeons of England*.

[16] Albucasis RF (2006). *(Abu Al-Qasim Al-Zahrawi): Renowned Muslim Surgeon of the Tenth Century*.

[17] Turgut M (2009). Surgical scalpel used in the treatment of “infantile hydrocephalus” by Al Zahrawi (936-1013 A.D.). *Child's Nervous System*.

[18] Annajjar J (2010). Abu Alkasem Al Zehrawi (Albucasis 936-1013). *Child's Nervous System*.

[19] Al-Rodhan NRF, Fox JL (1986). Al-Zahrawi and Arabian neurosurgery, 936-1013 AD. *Surgical Neurology*.

[20] Al-Ghazal SK (2007). *Al-Zahrawi (Albucasis) the Great Andalusian Surgeon*.

[21] San I, Oguz H, Kafali H (2005). Colored illustrations of pediatric otorhinolaryngologic surgical techniques of a Turkish surgeon, Serefeddin Sabuncuoglu, in the 15th century. *International Journal of Pediatric Otorhinolaryngology*.

[22] Gupta D, Bleakley B, Gupta RK (2007). Dragon’s blood: botany, chemistry and therapeutic uses. *Journal of Ethnopharmacology*.

[23] Dehbokri SG, Saeidiani S, Mohammadzadeh R, Gharabagh MS, Rezaeieh AA, Akradi L (2010). A comparative study of the healing effects of calendula and Astragalus fasciculifolius aqueous resin extract on rabbit skin wounds. *Journal of Veterinary Medical*.

[24] Mosaddegh M, Naghibi F, Moazzeni H, Pirani A, Esmaeili S (2012). Ethnobotanical survey of herbal remedies traditionally used in Kohghiluyeh va Boyer Ahmad province of Iran. *Journal of Ethnopharmacology*.

[25] Walsh ME, Reis D, Jones T (2010). Integrating complementary and alternative medicine: use of myrrh in wound management. *Journal of Vascular Nursing*.

[26] Wanner J, Schmidt E, Bail S (2010). Chemical composition and antibacterial activity of selected essential oils and some of their main compounds. *Natural Product Communications*.

[27] Fani M, Kohanteb J (2012). Inhibitory activity of Aloe vera gel on some clinically isolated cariogenic and periodontopathic bacteria. *Journal of Oral Science*.

[28] Dat AD, Poon F, Pham KB, Doust J (2012). Aloe vera for treating acute and chronic wounds. *Cochrane Database of Systematic Reviews*.

[29] Tambekar DH, Khante BS, Chandak BR, Titare AS, Boralkar SS, Aghadte SN (2009). Screening of antibacterial potentials of some medicinal plants from Melghat forest in India. *African Journal of Traditional, Complementary and Alternative Medicines*.

[30] Derkacz M, Chmiel-Perzyńska I, Nowakowski A (2011). Gynecomastia—a difficult diagnostic problem. *Endokrynologia Polska*.

[31] Diamandopoulos AA, Kassimatis TI, Goudas PC (2007). Paulus Aegineta: the pioneer of plastic surgery. Evolution and comparisons. *Hospital Chronicles*.

[32] Adams F (1844–1847). On male breasts resembling the female. *The Seven Books of Paulus Aegineta*.

[33] Dogan T, Bayramiçli M, Numanoglu A (1997). Plastic surgical techniques in the fifteenth century by Serafeddin Sabuncuoglu. *Plastic and Reconstructive Surgery*.

[34] Papadakis M, Manios A, De Bree E, Trompoukis C, Tsiftsis DD (2010). Gynaecomastia and scrotal rhacosis: two aesthetic surgical operations for men in Byzantine times. *Journal of Plastic, Reconstructive and Aesthetic Surgery*.

[35] Sharafkandi A (2008). *The Persian Translation of Qanoun Fi Al-Tibb (Or the Canon of Medicine)*.

[36] Hamarneh SK, Sonnedecker G (1963). *A Pharmaceutical View of Abulcasis Al-Zahrāwī in Moorish Spain*.

[37] Zakkour MY (2009). Index to explain the vocabulary contained in the text. *Kitab Al-Zahrawi Fi- Tibb Li Amal Al-Jarraheen (Or Al-Zahrawi’S Book in Medicine for SurgeonS)*.

[38] Oates SD, Singletary SE, Robb GL, Hortobagyi GN (2004). Gynecomastia. *Advanced Therapy of Breast Disease*.

[39] Arzani MA (2008). *TEbb-E-Akbari (Akbari’s Medicine)*.

[40] Klotz LO, Weser U, Rainsford KD, Milanino R, Sorenson JR, Velo GP (1998). Biological chemistry of copper compounds. *Copper and Zinc in Inflammatory and Degenerative Diseases*.

[41] Sorenson JRJ (1976). Copper chelates as possible active forms of the antiarthritic agents. *Journal of Medicinal Chemistry*.

[42] Nabipour I (2003). Clinical endocrinology in the Islamic Civilization in Iran. *International Journal of Endocrinology and Metabolism*.

